# *Streptococcus suis* in invasive human infections in Poland: clonality and determinants of virulence and antimicrobial resistance

**DOI:** 10.1007/s10096-016-2616-x

**Published:** 2016-03-15

**Authors:** A. Bojarska, E. Molska, K. Janas, A. Skoczyńska, E. Stefaniuk, W. Hryniewicz, E. Sadowy

**Affiliations:** Department of Molecular Microbiology, National Medicines Institute, Chełmska 30/34, 00-725 Warsaw, Poland; Department of Epidemiology and Clinical Microbiology, National Medicines Institute, Chełmska 30/34, 00-725 Warsaw, Poland; Department of Molecular Biology and Genetics, Gene Expression and Gene Medicine, C.F. Møllers Allé 3, building 1130, 422, 8000 Aarhus C, Denmark; Institute of Environmental Sciences, Jagiellonian University, Gronostajowa 7, 30-387 Cracow, Poland

## Abstract

The purpose of this study was to perform an analysis of *Streptococcus suis* human invasive isolates, collected in Poland by the National Reference Centre for Bacterial Meningitis. Isolates obtained from 21 patients during 2000–2013 were investigated by phenotypic tests, multilocus sequence typing (MLST), analysis of the TR9 locus from the multilocus variable number tandem repeat (VNTR) analysis (MLVA) scheme and pulsed-field gel electrophoresis (PFGE) of *Sma*I-digested DNA. Determinants of virulence and antimicrobial resistance were detected by polymerase chain reaction (PCR) and analysed by sequencing. All isolates represented sequence type 1 (ST1) and were suggested to be serotype 2. PFGE and analysis of the TR9 locus allowed the discrimination of four and 17 types, respectively. Most of the isolates were haemolysis- and DNase-positive, and around half of them formed biofilm. Genes encoding suilysin, extracellular protein factor, fibronectin-binding protein, muramidase-released protein, surface antigen one, enolase, serum opacity factor and pili were ubiquitous in the studied group, while none of the isolates carried sequences characteristic for the 89K pathogenicity island. All isolates were susceptible to penicillin, cefotaxime, imipenem, moxifloxacin, chloramphenicol, rifampicin, gentamicin, linezolid, vancomycin and daptomycin. Five isolates (24 %) were concomitantly non-susceptible to erythromycin, clindamycin and tetracycline, and harboured the *tet(O)* and *erm(B)* genes; for one isolate, *lsa(E)* and *lnu(B)* were additionally detected. *Streptococcus suis* isolated in Poland from human invasive infections belongs to a globally distributed clonal complex of this pathogen, enriched in virulence markers. This is the first report of the *lsa(E)* and *lnu(B)* resistance genes in *S. suis*.

**Electronic supplementary material:**

The online version of this article (doi:10.1007/s10096-016-2616-x) contains supplementary material, which is available to authorized users.

## Introduction

*Streptococcus suis* represents one of most important pathogens of pigs, responsible for septicaemia, meningitis, arthritis and pneumonia in newborn and young animals of this species [1]. These bacteria are also a cause of invasive diseases in humans, mainly meningitis, as well as septicaemia, endocarditis and arthritis [2]. Such infections are typically sporadic, and, in the majority of cases, occur in particular occupational groups, such as abattoir workers and butchers. Infection may also be acquired by contact with raw or undercooked meat products, traditionally consumed in the Far East of Asia [2] and, thus, *S. suis* should be considered a food-borne pathogen [3]. In some countries of this region, such as Vietnam, *S. suis* represents the most frequent cause of bacterial meningitis in adults [4]. *Streptococcus suis* of serotype 2 (SS2) is considered the most virulent in both humans and animals among the currently recognised 29 serotypes [2, 5]. Tonsil carriage of SS2 by healthy slaughterhouse pigs represents an important natural reservoir of this pathogen [6]. Other serotypes, sporadically isolated from humans, include 1, 4, 5, 14, 16, 21 and 24 [2]. The threat posed by *S. suis* to public health was further emphasised with reports of two outbreaks in China in 1998 and 2005, involving 25 and 215 patients, and 14 and 38 deaths, respectively [7]. Streptococcal toxic shock syndrome (STSS) and high mortality of patients, observed in both outbreaks, was attributed to the presence of the 89K putative pathogenicity island (89K PAI) found in strains responsible for these outbreaks [8]. Further studies identified genes encoding a two-component signal transduction system SalK/SalR, a type IV-like secretion system and a novel haemolysis-related gene *hhly3*, located within this element and presumably involved in the STSS development [9–11].

While *S. suis* shows quite significant variability of the general population structure, as revealed by e.g. multilocus sequence typing (MLST), human isolates belong almost exclusively to a single clonal complex (CC), CC1, with a central and likely ancestral sequence type 1 (ST1) associated with serotype 2 [12]. The most widely studied virulence-associated factors of *S. suis* include suilysin (Sly), extracellular factor (EF), fibronectin-binding protein (FBP), muramidase-released protein (MRP), surface antigen one (Sao), enolase (Eno), DNase (SsnA), serum opacity factor (OFS), pili and others [13–17]. Human isolates of *S. suis* retain susceptibility to penicillin, ceftriaxone and vancomycin, but are frequently resistant to tetracycline and erythromycin, e.g. a study in Vietnam showed prevalence rates of resistance to these compounds as high as 83 % and 20 %, respectively [4]. Integrative conjugative elements (ICE) seem to play an important role in the transmission of resistance determinants to this species, as demonstrated by genomic studies [18]. Plasmids are observed in *S. suis* as well [18, 19], but their role in resistance development remains as yet little studied. Moreover, a recent study [20] has shown that *S. suis* is capable of developing competence for DNA uptake in a process dependent on the *comR* and *comX* gene products, thus providing another possibility for the acquisition of resistance determinants.

Recently, a meningitis case due to *S. suis* was reported in Poland [21]. The diversity of *S. suis* strains involved in invasive human infections in our country, as well as their relationships to strains from Europe and other continents, remain unknown up to now. Therefore, we aimed at performing a detailed analysis of isolates, collected by the National Reference Centre for Bacterial Meningitis (NRCBM) located at the National Medicines Institute, in the respect of their phenotypic and genotypic features.

## Materials and methods

### Bacterial isolates and patient data

The NRCBM started its activity in 1997, and the first *S. suis* isolate from a human invasive infection was received in 2000. Between then and the end of 2013, 21 cases of invasive infection caused by *S. suis* (20 cases of meningitis and one case of endocarditis) were reported to the NRCBM from 13 hospitals located throughout Poland (Table 1). Seventeen (81 %) patients were male; the age ranged from 28 to 67 years (average, 50 years). Overall, 19 isolates were obtained from cerebrospinal fluid (CSF) and seven isolates were from blood. For five patients, isolates were received from both blood and CSF, but only one isolate from each patient was included in the analysis. Upon receipt, all isolates were re-identified using the Rapid ID32 STREP or the VITEK II GP system (both from bioMérieux, Marcy l’Etoile, France) and stored at −80 °C.

**Table 1:** Selected features of human invasive isolates of *Streptococcus suis* isolated in Poland from 2000 to 2013

Year	City^a^	Isolation material^b^	Antibiotic resistance profile ^c^	PFGE	TR9^d^	Haemolysis/*sly* gene^e,f^	Biofilm formation^f^	DNase activity^f,g^	OFS type	*sao* type	*mrp*, repeat region	*mrp*, variable 5′ region^h^	*ω-ε-ζ* ^f^	*repA* of ICE*Ssu*_SC84_ ICE*Ssu*_BM407_-2
2000	Gdańsk	CSF	S	A1	36	+++/+	neg	+	1	M	*mrp*	wt	neg	neg
2003	Gdańsk	CSF	S	A1	14	+++/+	pos	neg	1	M	*mrp*	wt	neg	neg
2004	Opole	CSF/blood	S	A1	35	++/+	neg	neg	1	M	*mrp*	wt	neg	neg
2004	Opole	CSF	S	A4	8	++/+	pos	+	1	M	*mrp*	wt	neg	neg
2005	Elbląg	CSF/blood	S	A1	35	++/+	pos	++	1	M	*mrp*	wt	neg	neg
2005	Kędzierzyn-Koźle	CSF	S	A1	42	++/+	neg	neg	1	M	*mrp*	wt	neg	neg
2006	Nowa Sól	blood^x^	S	A1	33	++/+	neg	+	1	M	*mrp*	wt	neg	neg
2007	Pleszew	CSF/blood	ECT	A1	15	++/+	pos	neg	1	6 repeats	*mrp*	Δ1699	neg	pos
2008	Gdańsk	CSF/blood	S	A1	28	neg/neg	pos	+	1	M	*mrp*	ins 571′	neg	neg
2009	Olsztyn	CSF	S	A1	15	++/+	neg	neg	1	M	*mrp* ^S^	wt	neg	neg
2010	Brzeg	blood^y^	S	A1	32	++/+	neg	++	1	M	*mrp*	wt	neg	neg
2010	Bełchatów	CSF	S	A1	34	++/+	neg	neg	1	M	*mrp*	wt	neg	neg
2010	Puławy	CSF	ECT	A3	19	++/+	neg	+	1	M	*mrp*	wt	pos	pos
2010	Koszalin	CSF	ECT	A1	26	++/+	pos	+	IS*Sag3*	M	*mrp*	wt	neg	pos
2011	Koszalin	CSF/blood	ECT	A2	72	+/+	pos	+	1	M	*mrp*	wt	neg	pos
2011	Chojnice	CSF	S	A1	29	++/+	pos	++	1	M	*mrp*	wt	neg	neg
2012	Elbląg	CSF	S	A1	26	++/+	pos	++	1	M	*mrp*	wt	neg	neg
2012	Wejherowo	CSF	S	A1	29	++/+	neg	++	1	M	*mrp*	wt	neg	neg
2013	Chojnice	CSF	S	A1	39	+/+	neg	++	1	M	*mrp*	ins 571′	neg	neg
2013	Koszalin	CSF	ECT	A1	61	++/+	neg	+	1	M	Δ162 bp	wt	neg	pos
2013	Opole	CSF	S	A1	23	+++/+	pos	+	1	M	*mrp*	wt	neg	neg

^a^City of patient hospitalization

^b^For five patients, two isolates were obtained (from CSF and blood), and both isolates shared all features tested

^c^S, susceptible to all antimicrobials tested; ECT, resistance to erythromycin, clindamycin and tetracycline (isolate harbouring the *lsa(E)*/*lnu(B)* genes underlined

^d^TR9, number of repeats in the TR9 locus from the MLVA scheme

^e^+, ++, +++, weak, intermediate and strong haemolysis, respectively

^f^pos, positive; neg, negative

^g^+, ++, weak and strong DNAse activity, respectively

^h^wt, wild-type sequence

^x^Patient diagnosed with endocarditis

^y^Patient diagnosed with meningitis

### Phenotypic studies

Antimicrobial susceptibility was tested using the broth microdilution method [22] for penicillin, cefotaxime, imipenem, erythromycin, moxifloxacin, tetracycline, chloramphenicol, rifampicin, gentamicin, linezolid and vancomycin; susceptibility to daptomycin was studied by the Etest method (bioMérieux, Marcy l’Etoile, France) and susceptibility to clindamycin by the disk diffusion method [22]. *Streptococcus pneumoniae* ATCC 46916 strain was used for quality control purposes. The results were interpreted following the breakpoints for viridans streptococci approved by the European Committee on Antimicrobial Susceptibility Testing (EUCAST) for penicillin, cefotaxime, imipenem, clindamycin, gentamicin and vancomycin, and the Clinical and Laboratory Standards Institute (CLSI) for of erythromycin, tetracycline, chloramphenicol, linezolid and daptomycin [22, 23]. In the case of moxifloxacin and rifampicin, *S. pneumoniae* breakpoints were used [23]. Haemolysis was evaluated visually as a distinct zone around bacterial colonies on Columbia agar with 5 % horse blood (bioMérieux, Marcy l’Etoile, France). The ability of isolates to form biofilm was evaluated in microtitre plates in BHI liquid medium with 0.5 % glucose and with or without 2.5 mg/ml of human plasma fibrinogen (Sigma-Aldrich, St. Louis, MO, USA), followed by staining with crystal violet, as previously described [24]. A biofilm-forming clinical isolate of *Enterococcus faecalis* from our collection was used as a positive control. The experiment was performed in triplicate and isolates with a mean OD_550_ ≥0.12 were considered positive in the test. DNase activity [17] was tested by direct visual evaluation on DNase agar with Methyl Green (Becton Dickinson, Sparks, MD, USA), using *Staphylococcus aureus* ATCC 25923 as a positive control.

### DNA isolation and bacterial typing

Total DNA was purified using the Genomic DNA Prep Plus kit following the manufacturer’s instructions (A&A Biotechnology, Gdynia, Poland). MLST was performed as previously described [12]; allele numbers and sequence types (STs) were assigned using the MLST database http://ssuis.mlst.net/ (accessed 17th December 2015). Pulsed-field gel electrophoresis (PFGE) analysis of *Sma*I-digested DNA was performed following the standard procedure [25]. The number of variable number tandem repeats (VNTR) (5′GAGCA)_n_ in the TR9 locus included in the proposed multilocus VNTR analysis (MLVA) scheme [26] was established using polymerase chain reaction (PCR) amplification of TR9 and sequencing of the products. Serotype of isolates was determined using primers specific for the *cps* loci of serotypes 2 and 1/2 [27].

### Analysis of virulence determinants

The *fbpS*, *epf*, *eno* and *sly* genes were detected as described by others [27–30]. The presence of *orfC* was investigated by PCR with primers 5′-AGATTGGGATGAACTGGTCG and 5′-AATAGCCGTATGACCTGCCA, specific for *orfB* and *orfC*, respectively (GenBank accession number AJ416308 [30]). The 89K PAI sequences were searched using primers CH3 and CH4, specific for unique sequences within the PAI [31]. Additionally, PCR with primers CH1 and CH6 [31], targeting sequences adjacent to this PAI, was used to exclude its presence in isolates negative in the previous reaction. The *ofs* and *sao* types were investigated as described [16, 32]. The pili genotypes were established using the published scheme employing primers specific for four pili gene clusters [15], including the sequencing of *sbp2*. The region encompassing 1–2286 bp in *mrp* was analysed by sequencing of the products of overlapping PCR [33], and the number of repeats in the repeat region by sizing of PCR products containing this region [34].

### Detection of antimicrobial resistance, transposon and plasmid genes

Tetracycline resistance genes *tet(M)*, *tet(O)*, *tet(L)*, *tet(K)*, *tet(W)*, macrolide resistance genes *erm(B)*, *mef(A)* and *int*_Tn916_ were detected by PCR as described by others [35–38]. For the detection of *tet(40)* and the lincosamide resistance genes *lsa(E)* and *lnu(B)*, primer pairs tet40-up/tet40-dn (5′-CTACCTGCTGTTCCGATTTGTC and 5′-TGATGAAGGTATCACCGCAACC), lsaE-up/lsaE-dn (5′-TATGCGTATTCCGGCAATATAAG and 5′-AACGGCTTCCTGATGTCTTG) and lnuB-up/lnuB-dn (5′-CGTGGGGAATTTCATTTCCTTTC and 5′-CGTTGATTCCCATCAACCATAG) were used, respectively. The linkage between genes *lsaE-lnuB* and *ermB-tetO* was investigated with primers lsaE-up/lnuB-dn and ermB-1/tetO-dn [36, 38], respectively. The presence of *rep1* and *rep2* genes, characteristic for broad-host range streptococcal and enterococcal plasmids, was tested using primers and conditions proposed in the Gram(+) plasmid typing scheme [39]. The *rep*_pBM407_ gene [18] was searched by PCR with primers rep407-up/rep407-dn (5′-GTATCGCACGTATTCCTCGTG and 5′-CATAATAGCCTTTTCCCCACGA). The *ω-ε-ζ* and *relBE* genes of plasmid toxin–antitoxin systems (TAS) were searched for as previously described [40]. The *repA* gene, specific for ICE*Ssu*_SC84_ and ICE*Ssu*_BM407_-2 [18], was detected with consensus primers repA_up/repA_dn (5′-TTAAGGTAGCCTACGCGGTTTTA and 5′-GTCGTCTCAGTTGCTTRGTCC). DNA from previously characterised clinical isolates of *Streptococcus agalactiae*, *E. faecalis* and *Enterococcus faecium* from our collection were used as positive controls for the detection of *tet(M)*, *tet(O)*, *tet(L)*, *tet(K)*, *tet(W)*, *erm(B)*, *mef(A)*, *int*_Tn916_, *rep1*, *rep2*, *ω-ε-ζ* and *relBE*. Competence-associated genes *comX* and *comR* were detected with primer pairs comX-up/comX-dn and comR-up/comR-dn (5′-GGATCGAGATGATTGGGAAC/5′-CATGTGGCATACGGTCAAAC and 5′-CTGAAGTTTGATGTGCTTCGC/5′-TTTCCAAAGCCTGCTGTACCT, respectively).

### DNA sequence analysis

Sequences were analysed using the Lasergene package (DNASTAR, Madison, WI, USA). New sequences found in this study were submitted to the GenBank: *sao* (KJ739797), *mrp* (KJ776426, KJ776427, KJ746998, KJ746999), *IS*Sag3-like (KR137531).

## Results

### Serotypes and clonality of isolates

PCR and MLST analyses demonstrated that all isolates were positive for serotypes 2- and 1/2-specific PCR and belonged to ST1. Because no human infection due to serotype 1/2 strains has yet been reported in any country [41], the PCR results strongly suggest that all the isolates are serotype 2. PFGE analysis identified four related patterns, A1–A4 (Table 1 and Supplementary Fig. 1). The predominant pattern, A1, was characteristic of 18 isolates. Sequencing of the TR9 locus from the MLVA scheme revealed 17 variants, with 8–72 repeats.

### Virulence phenotypes and determinants

Ten isolates (48 %) were able to form biofilm in the medium supplemented with fibrinogen (Table 1); no biofilm was observed in the absence of this compound. Fifteen isolates (71 %) showed variable levels of DNase activity. With a single exception, all isolates caused various degrees of β-haemolysis of horse blood and harboured the *sly* gene. This single isolate was negative in PCR for both *sly* and *orfC*. None of the isolates harboured sequences characteristic for the 89K PAI, and all isolates yielded expected ∼1.5 kb PCR product with primers specific for sequences located upstream and downstream of the PAI, further confirming its absence. All isolates carried the *fbpS*, *eno*, *epf*, *sao*, *mrp* and pili genes. The size of the PCR product for *epf* corresponded to the basic variant of this gene without insertions in the 3′ part [28]. The *sao* gene represented the M-type (i.e. with seven repeats) [32], with the exception of one isolate, which had six repeats. Analysis of the 3′ part of the *mrp* gene by PCR showed the presence of a single repeat for the majority (19) of isolates; one isolate had the *mrp*^S^ variant [34] and one had a novel *mrp* variant, containing a 162-bp deletion in the repeat region, encompassing nt4898–5059. Partial sequencing of the variable region of *mrp* revealed the presence of two new mutations, both resulting in a translational frameshift, a two-nucleotide insertion at position 571 in two isolates and a single-nucleotide deletion at position 1699 in one case. For the remaining isolates, the sequence was 100 % identical with the European variant (GenBank accession number X64450) of *mrp* [33]. Analysis of the pili gene clusters showed that all isolates belonged to genotype A, i.e. they were positive for the *srtBCD* cluster, the *srtE*/*sipE* genes and the *srtF* cluster. All isolates contained a single T insertion at position 798′ of the *sbp2* gene, which split the *sbp2* open reading frame (ORF) into *sbp2′* and *sbp2″* [15]. All isolates carried the type 1 *ofs* gene, with the exception of one isolate, for which partial sequencing revealed the presence of the IS*Sag3*-like insertion sequence from *S. agalactiae* [42] (97 % identity at the nucleotide level) at nt1165, in the same orientation as the *ofs* gene.

### Antimicrobial susceptibility profiles of isolates, resistance and competence determinants

All isolates were susceptible to penicillin, cefotaxime, imipenem, moxifloxacin, chloramphenicol, rifampicin, gentamicin, linezolid, vancomycin and daptomycin. Five isolates (24 %) were concomitantly resistant to tetracycline, erythromycin and clindamycin (Table 1). Resistant isolates had three different PFGE patterns (A1, A2, A3) and they all harboured the *tet(O)* and *erm(B)* genes; other tetracycline and macrolide resistance genes, such as *tet(M)*, *tet(40)*, *tet(L)*, *tet(K)*, *tet(W)* and *mef(A)* were not detected. None of the isolates was positive for the *ermB-tetO* linkage, characteristic for the ICE*Ssu*_BM407_-2 [18]. Additionally, one isolate was positive for the *lsa(E)* gene and the *lnu(B)* gene (in both cases verified by sequencing). As demonstrated by PCR with primers specific for the two genes, *lnu(B)* was located directly downstream of *lsa(E)*. All 21 isolates tested negative for the presence of transposon- and plasmid-specific genes, such as *int*_*Tn*916_, *rep1*, *rep2*, *rep*_pBM407_ and *relBE*; a single resistant isolate carried the *ω-ε-ζ* genes of TAS. The *repA* gene, characteristic for ICE*Ssu*_SC84_ and ICE*Ssu*_BM407_-2, occurred exclusively among five resistant isolates. All isolates were positive for the *comR* and *comX* competence genes.

## Discussion

*Streptococcus suis* is currently emerging as an important zoonotic pathogen in humans, especially in some regions of the world. The aim of our study was to provide extensive characterisation of isolates of this pathogen observed in Poland since 2000. Most of the patients affected by *S. suis* infection were male and middle-aged; the patient’s profession is not routinely reported to the NRCBM, but for two patients (a farmer and a butcher), infection was very likely caused by occupational exposure. Misidentification is considered a common cause for the underestimation of rates of invasive infections caused by *S. suis* [2]. In our study, isolates were reported to the reference laboratory as *S. suis* in only 11 cases (52 %); five isolates were reported as *Streptococcus* spp. and two as *Streptococcus bovis*, while the remaining three isolates were misidentified as *S. agalactiae*, *Streptococcus anginosus* and *Streptococcus sanguinis*.

On the basis of the serotype-specific PCR results and epidemiological information reported so far [41], all studied isolates were determined as serotype 2, which is most frequently observed in infections of humans [2, 41]. It has to be noted, however, that DNA-based methods do not allow discerning between serotypes 2 and 1/2, whose *cps* loci do not differ by any serotype-specific genes and whose sequences are almost identical [43]. Although strains of serotype 1/2 have not been reported from human infections [41], the presence of isolates with this serotype cannot be completely excluded in our collection due to the limitations of the method used. MLST uniformly identified all isolates as ST1. Isolates of this ST and its variants have been reported from human invasive infections worldwide, although STs from other clonal complexes have also been described, including, for example, ST20 in France and the Netherlands, STs 25 and 28 in Thailand and Japan, and ST25 in the United States (summarised in [41]). As shown recently in a Dutch study [44], the diversity of human isolates of *S. suis* appears generally much more limited in comparison to strains circulating in pigs in the same geographic area and a similar time span, and the vast majority of STs and serotypes found in pigs, a natural reservoir of this pathogen, have never been isolated from humans [41]. Our PFGE analysis discerned four different profiles and we additionally applied MLVA, which was suggested as a typing method allowing additional discrimination of strains, e.g. during an outbreak [26]. In the case of ST1, variability was reported only for the TR9 locus, which was selected for in our analyses. These yielded 17 variants with an even bigger range of the repeat number (8–72) than observed in the original study [26]. Such high diversity of a presumably quickly evolving typing marker is consistent with the sporadic character of all cases of human infections from which our isolates were obtained.

Biofilms play an important role in reducing bacterial susceptibility to antimicrobials and clearance by the host immune system. Formation of biofilm by *S. suis* required the presence of fibrinogen in the culture medium [24], as also seen in our study. We observed various levels of biofilm formation among our isolates, which was also reported by others for SS2 [45]. Some of our isolates lacked detectable DNase activity, in contrast to the findings of Haas and co-workers, who reported all ten ST1 strains in their study as DNase-positive [17]. Almost all isolates demonstrated haemolytic phenotype, associated with the presence of the *sly* gene. Sly, a pore-forming toxin, plays an important role in the pathogenesis of *S. suis* infection [46] and several studies reported all ST1 human isolates as positive for *sly* [47–50], in contrast to representatives of other clonal groups found in humans [49] and isolates from non-invasive infections and carriage in pigs [30, 34]. This gene is typically well conserved in *S. suis* and loss of haemolytic activity is usually due to the replacement of *sly* by *orfC* encoding a product of unknown function [30]; however, one of the isolates in the current study lacked both *sly* and *orfC*. Such isolates were also observed by others [48] and our searches of the available genomic sequences of *S. suis* in the GenBank (as of 29th January 2016) revealed that a single strain YS56 (GenBank accession number ALMY01000022) was negative for these two genes in the corresponding position of its genome.

The isolates analysed in our study carried genes of several virulence-associated factors other than *sly*, such as *mrp*, *epf*, *fbpS*, *eno*, *sao* and *ofs*. The basic variant of the *epf* gene appears to be, similarly to *sly*, a marker specific for invasive ST1 strains [47–50], while *mrp*, *fbpS*, *eno*, *sao* and *ofs* appear to be much more common in the whole *S. suis* population [29, 32, 47–51]. In agreement with other observations, our analysis also revealed variation in the genes of some of these virulence-associated determinants, including novel alleles of *mrp*, *sao* and *ofs*. The *mrp* gene with a single 411-bp repeat in its 3′ part, most common among our isolates, is typical for ST1; a shorter version, *mrp*^S^, present in one isolate, was also observed for this ST [49]. Larger variants of *mrp* occur among representatives of other CCs associated with serotype 2, such as ST29 [49] and other serotypes [34]. Three isolates from our collection harboured new indel mutations in *mrp*, preventing the full-length Mrp protein synthesis. Such mutations are relatively common in *S. suis* [33], and isolates positive for the gene but negative for the protein expression are frequently observed [33, 34, 50]. Variability in the *mrp* gene may be associated with a selective pressure from the immunological system of the host [34]. The *sao-*M (seven repeats), observed in our study for almost all isolates, is the most common variant among various serotypes of *S. suis* [32], and type 1 *ofs* characteristic for all but one isolate is typical for ST1 [16]. In our study, all isolates belonged to the genotype A of pili and harboured the characteristic frame-shift mutation in *sbp2*. At least four different pili loci exist in *S. suis*, and the combination of presence/absence of particular genes allowed distinguishing 12 genotypes, with genotype A being characteristic for ST1 isolates from human infections and diseased pigs [15]. We did not detect sequences specific for the 89K candidate PAI, found in highly virulent strains involved in two outbreaks in China and, as yet, not observed anywhere else [31]. In summary, inclusion of all the study isolates into ST1 and SS2, together with the observed high number of established and putative virulence factors, are consistent with the features of a specific genetic cluster of *S. suis*, associated with human meningitis [52], described also as the epidemic and highly virulent (E/HV) group, showing resistance to phagocytosis in vivo, thus allowing bacteria persistence at high concentrations in the animal mouse model, a pre-requisite for the development of an inflammatory reaction in the host [53].

Importantly, the investigated isolates of *S. suis* from our collection retained susceptibility to agents recommended in Poland for empirical therapy of community-acquired bacterial meningitis in adults, such as cefotaxime and vancomycin (http://www.koroun.edu.pl, accessed 17th December 2015). As yet, no resistance to these compounds has been reported for *S. suis* isolated from humans [54–56]. We observed concomitant resistance to erythromycin and tetracycline for 24 % of isolates. Resistance to tetracycline appears to be common, or even very common, in *S. suis* from human infections, reaching 100 % among 114 strains of ST7 in China [54], 100 % among 33 isolates in Hong Kong [55] and 89 % among 175 isolates in Vietnam, with a clear increase over the period 1997–2008 [56]. Resistance rates to erythromycin were 21 % and 22 % in Hong Kong and Vietnam, respectively [55, 56], similar to our observations. The acquisition of resistance determinants by *S. suis* occurs chiefly in its animal host under the selective pressure exerted by the use of antimicrobial agents in animal production. In Poland, according to the European Surveillance of Veterinary Antimicrobial Consumption (ESVAC) report for 2011, the sales of lincosamides, macrolides and tetracyclines for food-producing animals amounted to 4.1, 25.6 and 179.6 tonnes, respectively [57], and resistance to erythromycin and tetracycline among pig isolates reached 31 % and 64 %, respectively, in 2004 [58]. In our collection, isolates resistant to tetracycline and erythromycin/clindamycin carried the *tet(O)* and *erm(B)* genes. The presence of *tet(O)* was reported for a small number of human isolates from Europe and North America [54]. The *tet(M)* gene, associated with Tn*916*, was the most widespread determinant of tetracycline resistance in human *S. suis* in China and Vietnam [54, 56]. In Vietnam, *tet(O)* was the second most common gene responsible for tetracycline resistance; *tet(L)* and *tet(W)* were also observed in that study [56]. The *erm(B)* gene was present in 95 % of erythromycin-resistant human *S. suis* in Vietnam [56], while in Hong Kong, most of the isolates carried *mef(A)* [55]. Thus, there are obvious differences in the local epidemiology of tetracycline and erythromycin resistance genes among human *S. suis*. Additionally, one of the resistant isolates in our collection carried the *lsa(E)* and *lnu(B)* genes. The *lsa(E)* gene encodes an ABC transporter that confers resistance to lincosamides, streptogramin A and pleuromutilins (LS_A_P phenotype) by efflux of the drugs from the bacterial cell [59], while *lnu(B)* (formerly *linB*) inactivates lincosamides by adenylation [60]. These two genes are found in several Gram(+) bacteria, e.g. *E. faecalis* (AF408195), the C2944 strain of *S. aureus*, the SGB76 strain of *S. agalactiae*, the pDX5 plasmid of *E. faecium* and the Ery-11 strain of the pig pathogen *Erysipelothrix rhusiopathiae* [61–64], but, to our knowledge, have not yet been reported in *S. suis*. Our search of genomic sequences of 375 pig and human isolates of *S. suis* [65], deposited at the European Nucleotide Archive (http://www.ebi.ac.uk/ena, accessed 2nd February 2016), revealed the presence of *lsa(E)* and *lnu(B)* in four pig isolates, associated with serotype 2 from Vietnam and in two pig isolates of serotypes 7 and 14 from the United Kingdom, suggesting independent acquisition of these resistance genes by different clones in various parts of the world. The macrolide, lincosamide and tetracycline resistance determinants detected among isolates from our collection were likely carried by *S. suis*-specific ICEs [18], consistent with the concomitant presence of their *repA* gene and absence of genes characteristic for other mobile elements, such as Tn*916* and broad-host plasmids.

In conclusion, we performed a detailed phenotypic and genotypic analysis of *S. suis* isolates obtained in Poland from invasive human infections. While all these isolates belonged to a single clonal complex associated with considerable morbidity and mortality in humans worldwide, diversification of this complex was observed, including the presence of novel variants of virulence determinants and the acquisition of antimicrobial resistance genes. The human invasive *S. suis* infections in Poland very likely remain underestimated, considering the fact that, in 2012, the number of professionally active farmers amounted to 2.1 million and pig livestock exceeded 10 million heads (http://www.stat.gov.pl, accessed 17th December 2015), which indicates an existence of a significant at-risk human population and a need for improved surveillance of invasive human infections, caused by this pathogen in our country.

## Electronic supplementary material

Below is the link to the electronic supplementary material.

Supplementary Fig. 1 PFGE patterns among human isolates of *S. suis* in Poland. (DOCX 172 kb)
