# Supplementary material for: Streptococcus suis in invasive human infections in Poland: clonality and determinants of virulence and antimicrobial resistance
Source: Eur J Clin Microbiol Infect Dis. 2016 Mar 15;35:917–25. doi: 10.1007/s10096-016-2616-x (PMC4884564; doi:10.1007/s10096-016-2616-x)
Supplement: Supplementary file 1 — PFGE patterns among human isolates of S. suis in Poland. (DOCX 172 kb) [file 10096_2016_2616_MOESM1_ESM.docx]

Supplementary Fig. 1. PFGE patterns among human isolates of *S. suis* in Poland.


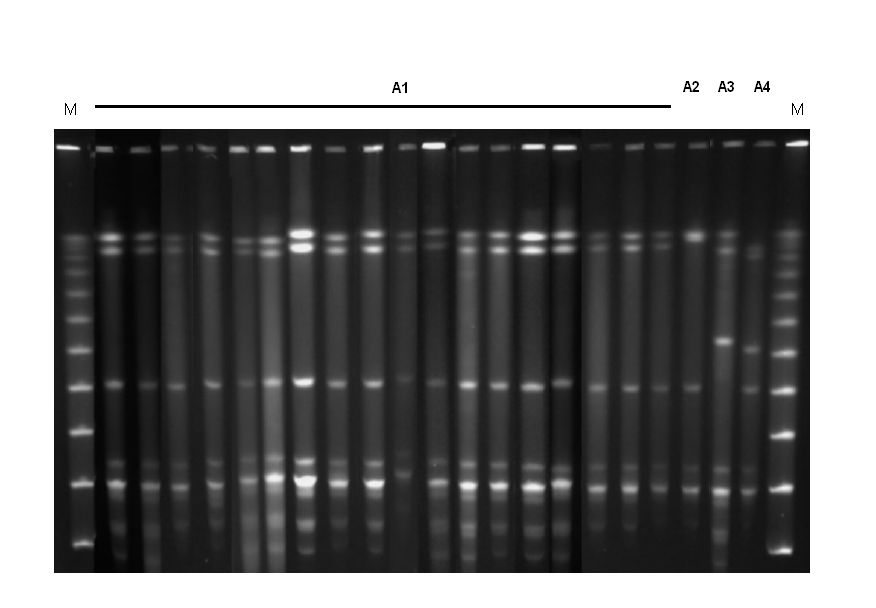


M, molecular weight marker (Lambda PFG Ladder, New England BioLabs, MA); A1-A4, PFGE patterns observed among 21 analysed isolates.
